# Supplementary material for: Caregiver burden in Buruli ulcer disease: Evidence from Ghana
Source: PLoS Negl Trop Dis. 2021 Jun 1;15(6):e0009454. doi: 10.1371/journal.pntd.0009454 (PMC8195390; doi:10.1371/journal.pntd.0009454)
Supplement: S1 Text — (PDF) [file pntd.0009454.s002.pdf]

Caregiver burden in Buruli ulcer disease

Topic guide

Target group:	
Caregivers	
Key topics:	
Caregiver routine responsibilities<br>Impact of BUD on daily life<br>Available support for caregiving role<br>Barriers/challenges in accessing healthcare	
Domain	Questions
Introduction/rapport	Introduce yourself:<br>-your name<br>-How old you are<br>-Community you reside<br>-Your highest level of education<br>-Your marital status<br>-Size of your household<br>-Number of sick BUD patients in your household<br>-Relationship to sick/former BUD patient
Caregiver routine responsibilities	Work related:<br>- The work you do<br>-Average number of hours (within a day or night) spent at work.<br>-How you cater for household (if there are other sources of income apart from work)<br>Sick patient related:<br>-What you do/did specifically for the sick BUD patient.<br>✓ Were you bathing and dressing him/ her?<br>✓ Were you cooking for him/ her?<br>✓ Were you running errands for the sick relative?<br>✓ Were you the one paying for feeding and accommodation for the sick patient?<br>✓ Were you the one providing for his/her medical care?<br>✓ Were you the one taking the sick person to the BUD clinic for management?<br>✓ Other responsibilities you took on for the sick person not discussed earlier
Impact of caregiving role on daily life	Impact on support for other family members<br>-has caregiving role affected support for other family members?

	✓ Quality time spent with other family members reduced?<br>✓ Provision of basic needs and accommodation<br>✓ Support for their health and well being<br>✓ Reduced support for their educational or work-related needs<br>-added roles for other family members<br>✓ Have other family members had to take on some of your responsibilities because of your caregiving role?<br>✓ Talk about your roles other family members have had to take on, for instance taking over domestic chores while you take the sick BUD patient to the hospital, going to the farm on your behalf, having to provide financial assistance since your output is low etc.<br>-Has there been any impact of the BUD on other aspects of the life of family members not discussed above?<br>✓ Has it impacted on the social life and interaction of family life? For instance. has it influenced how family members interact within their communities? (stigma)<br>✓ Has it impacted on their spiritual life?<br>✓ Has it impacted on their psychological health through worry?<br>Impact on caregiver<br>-How caregiving role has affected caregiver<br>✓ Has it impacted on work output of caregiver?<br>✓ Has it impacted on the social interactions and relationships?<br>✓ has it impacted on finances of caregiver?<br>✓ has it impacted on the psychological well-being?<br>✓ Has it impacted on the health of caregiver
Available support for caregiving role	-kind of support caregiver received<br>✓ did caregiver receive any financial support like money for transportation, feeding, provision of basic needs, medication etc.<br>✓ did caregiver receive physical support like transporting patient to hospital, taking care of responsibilities of caregiver while she/he attended to patient<br>✓ did caregiver receive emotional/psychological support for instance some form of encouragement, advice<br>✓ spiritual support in terms of praying for deity, assisting caregiver to seek divine intervention among others.

	✓ Other form of support caregiver received that was not discussed earlier.<br>-Groups of people that provided support for caregiver.<br>✓ Family- briefly discuss what they did<br>✓ Friends- briefly discuss what they did<br>✓ Neighbours- briefly discuss what they did<br>✓ Healthcare staff- briefly discuss what they did<br>✓ Colleagues- briefly discuss what they did
Barriers/challenges in accessing healthcare	Challenges encountered when accessing healthcare for sick patient<br>-Transportation that is,<br>✓ Distance from home to hospital<br>✓ means of moving to hospital (by walking, canoe, tricycles, vehicles etc)<br>✓ How readily accessible are the means of transport<br>-Finances<br>✓ Finances readily available to take patient to hospital?<br>✓ Finances to cover medical cost and other ancillary costs.<br>-Difficulty in mobility<br>✓ Could patient walk or needed to be carried to hospital.<br>-Any other barriers to accessing medical care.
