# Supplementary material for: Caregiver burden in Buruli ulcer disease: Evidence from Ghana
Source: PLoS Negl Trop Dis. 2021 Jun 1;15(6):e0009454. doi: 10.1371/journal.pntd.0009454 (PMC8195390; doi:10.1371/journal.pntd.0009454)
Supplement: Attachment — Submitted filename: Response to review comments_Caregiver burden in BUD.docx [file pntd.0009454.s003.docx]

30^th^ March 2021

**School of Medicine and Dentistry, Kwame Nkrumah University of Science and Technology (KNUST)**

**Kumasi, Ghana**

**The Editor**

**PLoS Neglected Tropical Diseases Journal**

Dear Sir,

**Re: "Caregiver burden in Buruli Ulcer Disease: evidence from Ghana"** **(PNTD-D-21-00059) - [EMID:e941025720146cc4]**

My co-authors and I are grateful to the Editor and reviewers for the useful comments on our above titled manuscript and wish to respond as follows:

Editorial comments
Thank-you for your submission and hard work in undertaking this study on Buruli ulcer disease.
Please note and address the helpful comments detailed by the reviewers below which I concur will improve the clarify of the manuscript.

Reviewer's Responses to Questions

Reviewer #1: Please see General Comments

Reviewer #2: This study is a qualitive study conducted in Ghana among 13 caregivers of BU patients. Majority of whom had a healed BU lesion. I think the study should be described as such to reflect that the caregivers are caregivers of former BUD. This should be indicated in the title. In addition the study did not include BUD patients with permanent disability. I would expect different perspective from caregivers of patients with permanent disability. Having said that the objectives of the study are clear, the study design is appropriate to respond to the objectives of the study. I have indicated my concern on the appropriateness of the study population for the study. I think this can be adjusted by revising the title of the study to include former BUD cases. The sample size was based on saturation which is appropriate for a qualitive study design. Proper ethical procures were followed.

Response:

We thank the reviewer for the comment. Indeed as indicated by the reviewer, the majority of participants in this study were caregivers of former patients but there was a participant who provided care for a patient with active BUD infection. It is therefore not the case that all participants were caregivers of former BUD patients. We believe stating in the title that participants were caregivers of former BUD patients will not be an accurate description of all the study participants. We think it best to keep the title ‘Caregiver burden in Buruli ulcer disease: evidence from Ghana’ but have added the following definitions in the methods section to provide further clarity:

‘Caregivers of former BUD patients (past infection) were defined as those who provided care for patients who were confirmed with BUD within the past 12 months and had completed antibiotic therapy with healed lesions. A caregiver for active BUD patient was one who provided care for a patient with confirmed BUD who was still receiving antibiotic therapy and whose lesion had not healed’.

Results
-Does the analysis presented match the analysis plan?
-Are the results clearly and completely presented?
-Are the figures (Tables, Images) of sufficient quality for clarity?

Reviewer #1: Please see General Comments

Reviewer #2: The results are presented appropriately. The analysis was also conducted clearly and following standard methods. My only comment here is since the number of participants is only 13, better to avoid percentage. Presenting the actual number would suffice.
Response: Done as suggested. All percentages have been deleted from the text and all results have been presented as proportions.

Conclusions
-Are the conclusions supported by the data presented?
-Are the limitations of analysis clearly described?
-Do the authors discuss how these data can be helpful to advance our understanding of the topic under study?
-Is public health relevance addressed?

Reviewer #1: Please see General Comments

Reviewer #2: The conclusions of the study are supported by the data presented in the study. The authors have discussed some of the limitations of the study, one example could be the have not discussed that they did not included caregivers of BUD with active lesion, which might have introduced bias to the study.
The public health relevance of the study is not well addressed. The authors should discuss how the study helps programme implementations.

Response:

It is not the case that the study did not include any caregiver of patient with active BUD lesion. While the majority of participants in this study were caregivers of former patients, there was a participant who provided care for a patient with active BUD infection (see also response to reviewer 2 above). We have further clarified by inserting the following in the methods section:

‘Caregivers of former BUD patients (past infection) were defined as those who provided care for patients who were confirmed with BUD within the past 12 months and had completed antibiotic therapy with healed lesions. A caregiver for active BUD patient was one who provided care for a patient with confirmed BUD who was still receiving antibiotic therapy and whose lesion had not healed’.

Further, we have included the following statement in the discussion section to highlight the public health relevance of our study’s findings:

‘The Sustainable Development Goals (SDG) target 5.4 seeks to recognise and value un/under paid care and domestic work. Caregivers of patients with chronic NTDs provide unpaid care. The BUD control programme in Ghana should ensure that there are pathways to provide advocacy for support of caregivers of persons with chronic NTDs like BUD. Caregivers should be included in the programmatic planning for management of patients with chronic NTDs like BUD. National BU control programmes should include packages like education and psychosocial support services that will help improve the experience of caregivers of patients.’

Editorial and Data Presentation Modifications?
Use this section for editorial suggestions as well as relatively minor modifications of existing data that would enhance clarity. If the only modifications needed are minor and/or editorial, you may wish to recommend “Minor Revision” or “Accept”.

Reviewer #1: Please see General Comments

Reviewer #2: (No Response)

Summary and General Comments

Reviewer #1: This is an interesting manuscript that expands the relatively small body of literature on the roles, activities and impacts on caregivers of supporting a person affected by a Neglected Tropical Disease (NTD). Amoako and colleagues have conducted a small qualitative study on caregivers of people with Buruli Ulcer disease (BUD) recruited at specific BUD clinics at three hospital sites in Ghana. They analyse data using themes identified in a recent qualitative study of caregivers of people with podoconiosis, another skin-NTD, and describe the financial, psychological, occupational and emotional impacts on the caregivers and other family members.
While overall, this is a useful contribution to the literature, I offer a number of comments that may increase the accuracy of description of the methods, and also improve the Discussion. Several of these arise from discrepancies between what is indicated in the COREQ checklist and what is found in the manuscript.
Methods
Participant recruitment (lines 98-110). Please include a statement as to whether any caregiver declined to participate, or dropped out. This is item 13 of the checklist, which you indicate has been addressed on p7, but I cannot find where.

Response: Done. We have inserted the sentence ‘No caregiver declined participation or dropped out from the study’.
Line 115. The study psychiatrist is referred to as ‘JP’, but I cannot find a name on the author list whose initials these might be.

Response: We have corrected the psychiatrist’s initials to read JPO.
Data collection (lines 112-121). You need to add who conducted the interviews, their qualifications and gender (as per items 1-5 on the COREQ checklist).

Response: Done as required. We have inserted in the methods section the sentences ‘The interviews were conducted by 2 male medical doctors with several years of clinical and research experience [YAA (MD), JPO (MD)] and 2 female researchers [NA (MPhil), AGO-A (MD)].’
Line 119. 20-40 minutes does seem short for qualitative interviews based on a topic guide. Please attach the topic guide to your revision to make it easier to assess whether sufficient depth was likely to have been reached.

Response: Interviews lasted an average of 60 minutes. This has been corrected in the manuscript
Data analysis (lines 132-143). There seems to be some confusion between transcription and translation in this para, eg line 132 “Interviews were first transcribed to Microsoft Word in English” – do you mean in Twi? Lines 133-4 “The transcripts were then read…and verbally translated into English..” – this presumably means the transcripts had been in Twi. Presumably the translations were written as well as ‘verbal’? Please clarify this para.

Response: We have revised the section to bring clarity. It now reads ‘The interviews (conducted in the local language, Twi) were translated and transcribed into English from the audio recordings by the study coordinator (NA).’
Either under Methods or in the Discussion (as a limitation), you need a statement about the biases and assumptions of the interviewers and those analysing the data (item 8 of the COREQ checklist).
Response: We have inserted the statement ‘The interviewers have been involved in providing care for BUD patients who attend the network of clinics where participants were recruited.’
Results
The choice of quotes is good, and there is no undue repetition.
Lines 148-9. Could you clarify this - do you mean caregivers had previously given care to patients whose lesions had healed completely in addition to the patient they accompanied to hospital, or that the patient they accompanied to hospital had a lesion that had healed completely (in which case why did they need to go to hospital)?

Response: We have clarified the section by inserting the following statements in the methods section:

‘Caregivers of former BUD patients (past infection) were defined as those who provided care for patients who were confirmed with BUD within the past 12 months and had completed antibiotic therapy with healed lesions. A caregiver for active BUD patient was one who provided care for a patient with confirmed BUD who was still receiving antibiotic therapy and whose lesion had not healed.’

In our BUD clinics, patients are followed up till 1 year after diagnosis even if their lesions are healed. Recruitment was done within this period in accordance with the definition of ‘caregivers of former BUD patients’ used in this study.
Line 182. Figure 1 is very blurred, please improve the resolution. Also add ‘adapted from (24)’.
Response: Done as suggested
Discussion
Line 325. Explain why you used these four themes.

Response: This categorization was based on the themes that emerged from the answers provided by study respondents.

Line 333. In discussing reduced caregiver occupational output, you might want to make comparisons with: “Quantifying the socio-economic impact of leg lymphoedema on patient caregivers in a lymphatic filariasis and podoconiosis co-endemic district of Ethiopia” (PLoS NTDs, <https://doi.org/10.1371/journal.pntd.0008058>)
Response: We have inserted in the discussion section the sentences:

‘In a study on podoconiosis patients’ caregivers in Ethiopia, caregivers provided care for an average of 14.8 and 15.5 days per month for patients with mild/moderate and severe disease respectively. Furthermore, the hardship experienced by family units was not only due to the patients decreased working hours, but, was also due to caregivers’ lack of productivity while providing care (32).’

Line 342. In the results, you say that only one caregiver reported stigmatising experiences, but in the Discussion you say “caregivers also faced the issue of stigma…” - which is correct? If only one caregiver did report this experience, do you think saturation was genuinely reached?

Response: We thank the reviewer for noting this. The text in the discussion is correct. We have modified the results section accordingly to reflect that stigma was experienced by multiple caregivers.

Lines 388-9. While it is good you are suggesting some practical measures, is providing financial assistance for patients really 'simple'?
Response: We agree with the reviewer that providing financial assistance is not simple. However, an approaching that uses public-private partnerships might help in this regard. We have revised the section to bring further clarity by stating:

‘Efforts should be made to provide financial assistance such as waivers on hospital care costs and other incentives for caregivers of BUD patients as this could go a long way to ease the burden of caregiving for BUD in Ghana.’

We also added further addressed the issue in the discussion as follows:

‘The Sustainable Development Goals (SDG) target 5.4 seeks to recognise and value un/under paid care and domestic work. Caregivers of patients with chronic NTDs provide unpaid care. The BUD control programme in Ghana should ensure that there are pathways to provide advocacy for support of caregivers of persons with chronic NTDs like BUD. Caregivers should be included in the programmatic planning for management of patients with chronic NTDs like BUD. National BU control programmes should include packages like education and psychosocial support services that will help improve the experience of caregivers of patients.’

Minor Issues & Typographic Errors.
Throughout – I prefer ‘data’ to be considered a plural noun (and so take a plural verb), but realise that not all journals take issue with it being used as a singular noun.
Line 92. ‘among’ or ‘with’ would be better than ‘on’.

Response: Done
Lines 114 & 116. ‘topic’ guides, not ‘topical’ guides!

Response: Done
Line 146. ‘The demographic characteristics of study participants are presented..’ (not ‘is’)

Response: Done
Line 187. Should read ‘..resulting in some cases, in them being requiring to sell off...'

Response: Done
Line 324. Remove second ‘in’.

Response: Done
Line 341. The rest of this sentence uses the past tense, so suggest ‘placed’ rather than ‘places’.

Response: Done
Line 370. Use ‘include’ rather than ‘have’.
Response: Done
Reviewer #2:

•    The abstract in the online system and in the manuscript are different align this two.

Response: Done
•    The authors indicated that ‘All but one (12 of 13) of the participants were caregivers of former BUD patients whose lesions had healed completely with no functional impairments.’ This should be described properly, how long has been since the lesion had been held. I am asking this because it is obvious that people would forget the burden of the care or at least they will not describe the burden properly as those who are currently caring for BU patient. In addition the duration of when the lesion has been healed would also affect, what the caregivers can remember and report. Therefore, this should be clearly discussed in the context and the implication on the findings should be discussed.

Response: See response to reviewer 1 above. We have inserted the sentences:

‘Caregivers of former BUD patients or patients with past infection were defined as those who provided care for patients who were confirmed with BUD within the past 12 months and had completed antibiotic therapy with healed lesions. A caregiver for active BUD patients was one who provided care for a patient with confirmed BUD who was still receiving antibiotic therapy and whose lesion had not healed’. This could have resulted in recall bias and we have acknowledged this in the study limitations by inserting ‘Furthermore the assessed caregiver burden could have been impacted by recall bias as most participants were caregivers of former patients’.

•    Since the number of participants is only 13, I do not think it is proper to use percentage. It would be better to reports the numbers in the text and table 1.

Response: Done. We have reported only the number rather than percentages.
•    Line 366: Can you please describe what ‘the cost of hospital care and transportation’ are?  It would be good to estimate the average duration of hospital stay of a BU patient to bring the discussion into context?

Response: We have inserted in the discussion section the sentences:

‘Hospital care costs may include direct costs like admission fees (when needed as may occur if sepsis develops from secondary wound infection), wound dressings, fees for surgical procedures like debridement and skin grafting (when done), feeding and indirect costs related to loss of economic activity. Most BUD care provided in Ghana is on outpatient basis but patients and caregivers may still have to cater for transportation to and from health facilities, feeding and other ancillary costs.’

Do you want your identity to be public for this peer review?</b> For information about this choice, including consent withdrawal, please see our <a href="<https://www.plos.org/privacy-policy>" target="_blank">Privacy Policy</a>.

Reviewer #1: No

Reviewer #2: No


My co-authors and I are grateful to the Editorial team and reviewers for the useful comments.

Yours Sincerely,

Yaw Ampem Amoako

Corresponding author
